# Estimation of the unemployment rate in Turkey: A comparison of the ARIMA and machine learning models including Covid-19 pandemic periods

**DOI:** 10.1016/j.heliyon.2023.e12796

**Published:** 2023-01-07

**Authors:** Dilek Surekci Yamacli, Serhan Yamacli

**Affiliations:** a Nuh Naci Yazgan Univ., Dept. of Economics, 38090, Kayseri, Turkey; b Nuh Naci Yazgan Univ., Dept. of Electr.-Electron. Engineering, 38090, Kayseri, Turkey

**Keywords:** Unemployment rate, ARIMA, ANN, Estimation

## Abstract

The article focuses on analyzing the robustness of Auto Regressive Integrated Moving Average (ARIMA) and Artificial Neural Networks (ANN) methods in unemployment rate estimation. In this context, a stochastic trend in the unemployment rate was determined by using monthly data in Turkey. The oil price, real exchange rate, interest rate and unemployment rate variables are imported into the ARIMA and ANN models with 176 data samples for the period of 01.01.2008-31.08.2022. The results of the conventional linear ARIMA and nonlinear ANN regressor models are compared. The comparison results show that the ARMA (2,1) model is the most suitable model for the unemployment rate estimation. This conclusion was reached based on ARMA (2,1) and ANN's RMSE, MAE, MAPE and R^2^ parameters. From the results of the specified criteria, it was found that both models gave results close to the actual unemployment rate however ARMA (2,1) was the more appropriate model for the current data set. The actual unemployment data and the estimated values are also given verifying the better modeling of the developed ARMA (2,1) model. In addition, there are meaningful relationships between month variables and the employment rate. This result supports that the unemployment possesses chronic reasons in Turkey. On the other side, the unemployment rate forecasting error of the ARMA (2,1) is higher than the ANN model for the 2020–2021 period during the intense pandemic. This result is important because it shows that during the times of the economic uncertainty caused by the Covid-19 pandemic, forecasts employing the neural network model is observed to have lower errors than the results of autoregressive moving average model. Therefore, under an economic uncertainty, it is shown that modeling the unemployment rate using artificial neural network provides novel insights for economic forecasting.

## 1 Introduction

Unemployment is an important problem all over the world, especially in emerging economies. From a macroeconomic point of view, unemployment leads to the loss of production causing a negative output gap [1]. Negative output gap means that the experiencing countries obtain a lower production level compared to their potential. High unemployment rate may cause contraction, recession or depression in these economies [2]. This study explores methods that contribute to the accurate and reliable estimation of the unemployment rate. The differences of the economic conditions of various countries obviously require diverse modelling approaches. For example, some countries produce technology causing limited demand for labor while labor-intensive countries have higher labor demands. The free market mechanism does not work well in some other countries and the problem of asymmetric information between the wages and the labor market and the related unemployment problem may happen [3]. On the other hand, there exist differences between oil producer and consumer countries leading to different demands for the modeling of the unemployment rate. In this paper, the robustness of the autoregressive integrated moving average (ARIMA) and artificial neural networks (ANN) in estimating the unemployment rate for Turkey as an emerging economy example are analyzed and the oil prices, real exchange rate, interest rate and time variables are considered as regressors.

The effects of the oil prices, interest rate and exchange rate on the unemployment rate has been extensively studied in the literature however, limited number of studies considered both ARIMA and ANN models. For example, a new study modelled unemployment rate using FARIMA/GARCH, FARIMA and machine learning techniques [4]. They have concluded that the FARIMA analysis is better for the estimation of the unemployment. Another study used a hybrid approach for the modelling of unemployment in Canada, Germany, Japan, Netherlands, New Zealand, Sweden, and Switzerland [5]. Their results show that hybrid modelling techniques provide better results compared to the conventional methods. A machine learning based approach was also used to model the unemployment rate in France, Spain, Belgium, Turkey, Italy and Germany using ARIMA, ANN and SVM modelling [6]. They have concluded that the conventional modelling techniques suits better for Spain while ANN and ARIMA based hybrid methods are recommended for other countries. Unemployment in the European Union is estimated using three machine learning methods namely decision trees (DT), random forests (RF), and support vector machines (SVM) in the study of [7]. The results of their study imply that the optimal RF model outperforms the other models by reaching a forecasting accuracy of 88.5% [7]. ARIMA is performed for the Indian youth in a recent study [8]. According to this study, ARIMA model performs better than the other models on the basis of forecasting accuracy via time series cross-validation. The unemployment rate in Mediterranean countries is also modelled using machine learning models in Ref. [9]. This study shows that the nonlinearity of the data is better captured by ANN models compared to the traditional time series models such as FARIMA. From a conventional models perspective, Uri and Boyd studied whether there is a relationship between crude oil prices and employment rate in the United States using Granger causality [10]. Similarly, Hooker questioned the relationship between the oil prices and unemployment rate in Granger cause context [11]. Uri has concluded that there is an empirical relationship between the crude oil prices and unemployment rate utilizing cointegration tests [12]. Another study considered the effects of both oil prices and the interest rate on unemployment using Toda-Yamamoto method and concluded that real oil price and real interest rate affects unemployment rate in the long run [13]. Karlsson and Shukur have also taken oil prices and interest rate as inputs to model unemployment rate, in this case for Norway [14]. They have concluded that the causal relationships between oil price, interest rate and unemployment rate increase as the time range increases. Nusair has studied the effects of the oil price and interest rate on unemployment rates in the United States and Canada and has shown that there is a real long-run relationship between these input variables (oil price and unemployment rate) and output variable (unemployment) via cointegration tests [15]. Cuestas and Gil-Alana had a unique approach to investigate the relationship between the oil prices and unemployment for Central and Eastern Europe [16]. They have concluded that the sign of oil price shocks and unemployment rate are in the same direction. Ordonez et al. also investigated the effects of the oil price shock on employment in Spain and they have exposed that the relationship between these two parameters are different in crisis and recovery phases [17]. Cuestas and Ordonez have also studied the correlation of oil prices and unemployment in the United Kingdom and concluded that their relationship is different before and after the start of the 2008 recession [18]. The long-term and short-term relationships between oil prices and employment in top oil producing areas in the United States are investigated by Michieka and Gearhart and they found that there is a long-term relationship between these two parameters [19]. In another study, the effects of oil prices, oil price uncertainty and interest rate on the unemployment rate in the United States are analyzed by a cointegration method and it is exposed that all of these three parameters have asymmetric effects on the unemployment rate [20]. In a similar, study, it is shown that oil price uncertainty affects unemployment rate in the United States based on a generalized autoregressive conditional heteroscedasticity (GARCH) model [21]. The relationship between the unemployment rate and the exchange rate has also been studied in the literature. For example, Feldman has shown that the volatility in the exchange rate increases the unemployment rate in industrial countries [22]. Frenkel and Ros have studied the effect of the real exchange rate on the unemployment rate for several Latin American countries [23]. On the other hand, interest rate also affects the unemployment rate as pointed out by Prag [24].

The objective of this paper is to analyze the robustness of Auto Regressive Integrated Moving Average (ARIMA) and Artificial Neural Networks (ANN) methods in unemployment rate estimation. In this context, a stochastic trend in the unemployment rate was determined by using monthly data for the period of 01.01.2008-31.08.2022 in Turkey. Dependent variable is the unemployment rate while the oil price, real exchange rate, interest rate and time variables for 12 months are included in the ARIMA and ANN models as regressors. The results of the conventional linear ARIMA and nonlinear ANN regressor models are compared in this study as there is a gap in the literature on the comparison of the performances of these models for the modelling of the unemployment rate. ARIMA (2,0,1) that it is ARMA (2,1) and ANN methods are utilized for modelling the unemployment rate and their results are compared for the Covid-19 pandemic and other time frames. An automatic lag selection ARMA (2,1) model is shown to have the mean absolute error (MAE) value of 0.260. On the other hand, a feedforward ANN with tangent hyperbolic activation functions is developed with resilient backpropagation heuristic training to model the effects of the oil prices, the interest rate and the exchange rate on the unemployment rate with a MAE value of 0.718. It is then concluded that ARMA (2,1) and ANN models have comparable accuracies while ARMA (2,1) model provides better insight into the effects of the exogenous variables on the unemployment rate.

This paper is organized as follows: the details of the considered dependent and independent variables, the ARIMA method, the stationarity test results and the developed ARIMA and ANN models together with their coefficients are given in the Materials and Methods section. Then, the forecasting performances of the ARIMA and ANN models, their plots, computation of the performance metrics and the comparison with the results existing in the literature are presented in the Results and Discussion section. The study is concluded by the interpretation of the forecasting performances of the developed models before, during and after the pandemic in the Conclusions section.

## 2 Materials and methods

The unemployment rate (*unemp*) used in the study is calculated as the ratio of the number of unemployed to the labor force taken as percentage [25]. Other regressor variables are the oil prices (*oil*), interest rate (*int*) and real exchange rate (*rexc*). All data are taken from The Central Bank Republic of Turkey (CBRT) for the date range from 01.01.2008 to 31.08.2022 [26]. The data after 2020 is included on purpose for including the effects of the pandemic and post-pandemic economics. Interest rate data is used as 2–14 days repo interest rate which represents the short time interest rate in Turkey. European Brent oil price (barrel, dollar) is used as oil prices data. Exchange rate indicator is used as the real exchange rate index which is deflated by the consumer price index (CPI) with the base year of 2003. In addition, all data are seasonally adjusted using the moving average method [27].

In order to use the ARIMA method, the dependent variable must not be stationary at the level [28]. In this context, it is necessary to examine the stationarity structures of both the dependent variable and all the variables used as regressors in the model estimation. In this sense, before the ARIMA analysis, the stability tests of the variables were performed as the first step. Augmented Dickey-Fuller (ADF), Phillips-Perron (PP) and Kwiatkowski–Phillips–Schmidt–Shin (KPSS) tests are utilized to check their stationarities [[29], [30], [31]]. The results of these tests are summarized in Table 1.

**Table 1:** Results of the ADF, PP and KPSS stationarity tests.

Tests/Variables			Unemp	Oil	Rexc	Int
ADF	Level	Intercept Prob. t stat.	−1.94 0.313 −3.468	−2.693 0.178 −2.576	−0.071 0.950 −3.468	−2.518 0.113 −3.468
		Trend &Intercept Prob. t stat.	−1.807 0.697 −4.011	−2.738 0.222 −4.012	−2.632 0.267 −4.012	−3.129 0.103 −4.012
	1st Difference	Intercept Prob. t stat.	−12.072 0.000 −3.468	−9.664^a^ 0.000 −3.468	−10.986^a^ 0.000 −3.468	−5.312^a^ 0.000 −3468
		Trend &Intercept Prob. t stat.	−12.058^a^ 0.000 −4.012	−9.648^a^ 0.000 −4.012	−11.006^a^ 0.000 −4.012	−5.314^a^ 0.000 −4.012
PP	Level	Intercept Prob. t stat.	−2.184 0.213 −3.468	−2.196 0.209 −3.468	−0.456 0.895 −3.468	−2.159 0.222 −3.468
		Trend &Intercept Prob. t stat.	−2.112 0.535 −4.011	−2.170 0.502 −4.011	−2.590 0.285 −4.011	−2.693 0240 −4.011
	1st Difference	Intercept Prob. t stat.	−12.129^a^ 0.000 −3.468	−9.418^a^ 0.000 −3.468	−9.608^a^ 0.000 −3.468	−7.684^a^ 0.000 −3.468
		Trend &Intercept Prob. t stat.	−12.114^a^ 0.000 −4.012	−9.326^a^ 0.000 −4.012	−9.652^a^ 0.000 −4.012	−7.692^a^ 0.000 −4.012
KPSS	Level	Intercept Prob. t stat.	0.502 0.463	0.466 0.462	1.577 0.463	0.629 0.463
		Trend &Intercept Prob. t stat.	0.184 0.146	0.313 0.146	0.305 0.146	0.192 0.146
	1st Difference	Intercept Prob. t stat.	0.089^a^ 0.463	0.071^a^ 0.463	0.062^a^ 0.463	0.093^a^ 0.463
		Trend &Intercept Prob. t stat.	0.077^a^ 0.146	0.049^a^ 0.015	0.035^a^ 0.146	0.056^a^ 0.146

a Indicates significance at 1% significance level.

Table 1 shows that per the results of the ADF, KPSS and PP stationarity tests, all variables are observed to have stationarities at their first-difference levels. As the second step, the data between 2008:01 and 2022:08 are taken as the training data and used in the ARIMA model with automatic lag selection. ARIMA is a linear time series model used in stationary time series results for the explanation of the linear propensity.

An ARIMA model is defined using three numbers as ARIMA (p, d, q) where p and q show the orders of the autoregressive and moving average parts, respectively and d is the differencing level [32]. An ARIMA model can be expressed as in Eq. (1).

(1) $$y_{t}=c+\sum_{i=1}^{p}\varphi_{i}x_{t-i}+\sum_{j=1}^{q}\theta_{j}\varepsilon_{t-j}+\varepsilon_{t}$$

where *y*_*t*_ demonstrates the data on which ARIMA model is applied, *x*_*t*_ are the differences series with the order of *d*, φ_i_ are the coefficients of the autoregressive terms, φ_j_ are the parameters of the moving average part and ε_t_ are the error terms. The lag selections are determined as AR(2)-MA(1), in other words p = 2 and q = 1 are obtained as a result of the automatic ARIMA forecasting method at the Eviews 10.0 software [33]. The ARIMA model with explanatory variables is developed in Eviews and the differencing level is determined as zero implying that ARIMA model is reduced to an ARMA model. The coefficients of the ARMA (2,1) model are obtained as in Table 2. According to Table 2, the coefficients of oil prices, exchange rate and interest rate variables are not statistically significant. All the coefficients obtained for each month of the 12-month period are statistically significant. This result reveals that it is important to consider monthly effects in modeling unemployment. Significant monthly effects provide clues in evaluating unemployment as a structural problem. The unemployment rate supported the ARMA (2,1) process. The R^2^ value of the model is 0.82.

**Table 2:** The coefficients of the developed ARMA (2,1) model.(2)$y_{t}=f\left(\sum w_{i}x_{t}+b_{j}\right)$

Variable	Coefficient	Std. Error	t-Statistic	Prob.
D(REXC)	−0.003	0.008	−0.404	0.6869
D(INT)	0.005	0.028	0.173	0.8627
D(OIL)	0.001	0.003	0.318	0.7513
@MONTH = 1	11.970	0.216	55.540	0.0000
@MONTH = 2	11.839	0.219	54.173	0.0000
@MONTH = 3	11.010	0.217	50.707	0.0000
@MONTH = 4	10.071	0.216	46.605	0.0000
@MONTH = 5	9.489	0.214	44.364	0.0000
@MONTH = 6	9.761	0.211	46.249	0.0000
@MONTH = 7	10.108	0.209	48.396	0.0000
@MONTH = 8	10.322	0.227	45.446	0.0000
@MONTH = 9	10.429	0.227	45.892	0.0000
@MONTH = 10	10.487	0.216	48.501	0.0000
@MONTH = 11	10.464	0.214	48.964	0.0000
@MONTH = 12	11.122	0.213	52.222	0.0000
AR(1)	1.887	0.069	27.282	0.0000
AR(2)	−0.913	0.0686	−13.306	0.0000
SAR(12)	1.746	0.098	17.802	0.0000
SAR(24)	−0.947	0.129	−7.339	0.0000
MA(1)	−0.698	0.122	−5.715	0.0000
SMA(12)	−1.933	1451.948	−0.001	0.9989
SMA(24)	1.000	1502.067	0.001	0.9995
SIGMASQ	0.058	43.67	0.001	0.9989

As the next step, the time series data is modelled using neural network. The artificial neural networks and optimization algorithms are used in a wide range of applications such as scheduling [34], routing [35] and outsource planning problems [36]. The ANNs can also be used as a fitting or regression tool for the mapping and forecasting data sets. A general single-output feedforward ANN model can be represented using Eq. (2).

In Eq. (2), *y*_*t*_ is the output series, *x*_*t*_ are the series representing exogenous variables, *w*_*i*_ are the node gains and *b*_*j*_ denote the additive terms. The function f() is called the activation function, which adds nonlinearity to the ANN model, which is usually selected as a sigmoid or tanh function in practice.

**Fig. 1:**
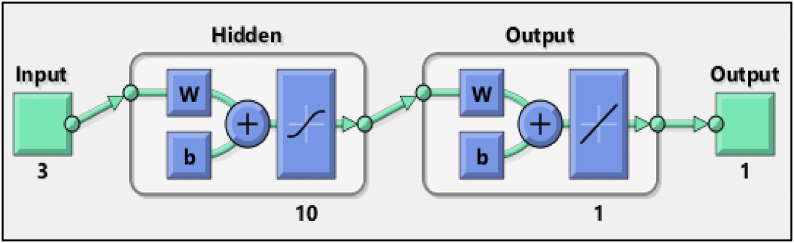
The feedforward neural network architecture used for the modeling in this study.

In this study, an ANN with 2 layers is used to realize the regression of the unemployment rate as a function of the oil prices, exchange rate and the interest rate as shown in Fig. 1. The number of hidden neurons is a critical parameter for the performance of the artificial neural network model, which directly influences the mean absolute error and the related parameters of the developed model. The number of hidden neurons is determined as 10 according to the minimization of the mean absolute error of the model in this study.

**Fig. 2:**
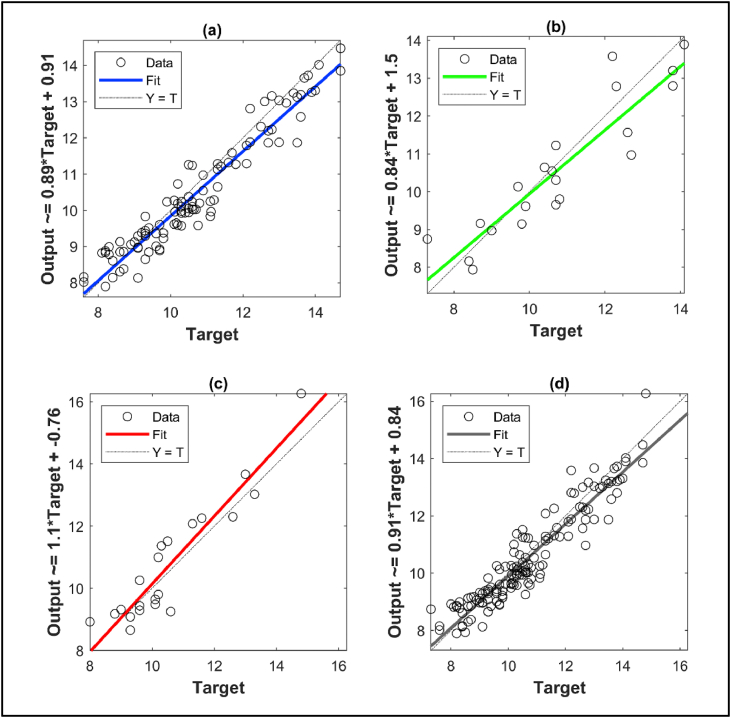
The plots showing the performances of the (a) training phase (b) validation phase, (c) test phase and (d) whole data regression.

**Fig. 3:**
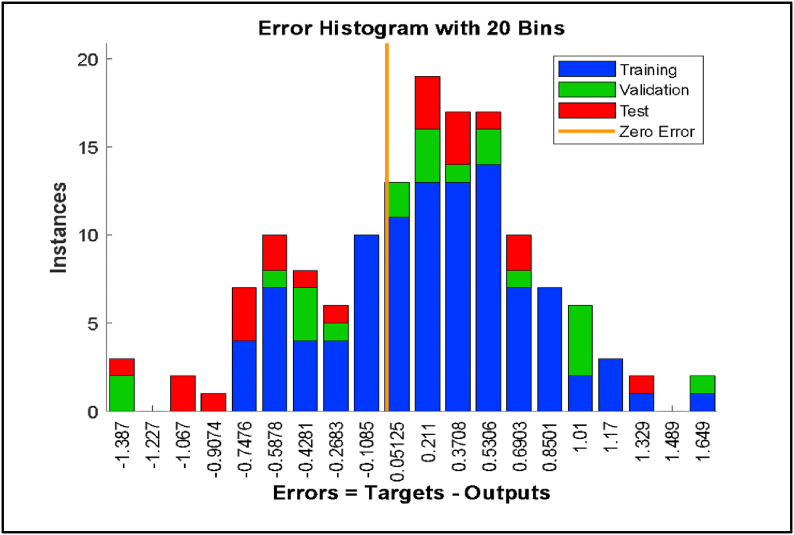
The error histogram of the ANN based modelling.

The resilient backpropagation algorithm is used for training the developed neural network as the activation functions are selected as tangent sigmoid as a general function [37]. 70% of the data are reserved for training the neural network while the test and validation data ratio is 30%. The MATLAB code and the coefficients of this neural network can be accessed from the link given at the end of the paper. The regression plots obtained after the training of the neural network are shown in Fig. 2 for the training data, the validation data, test data and the whole data. The error histogram of the ANN model is also shown in Fig. 3.

## 3 Results and discussion

**Fig. 4:**
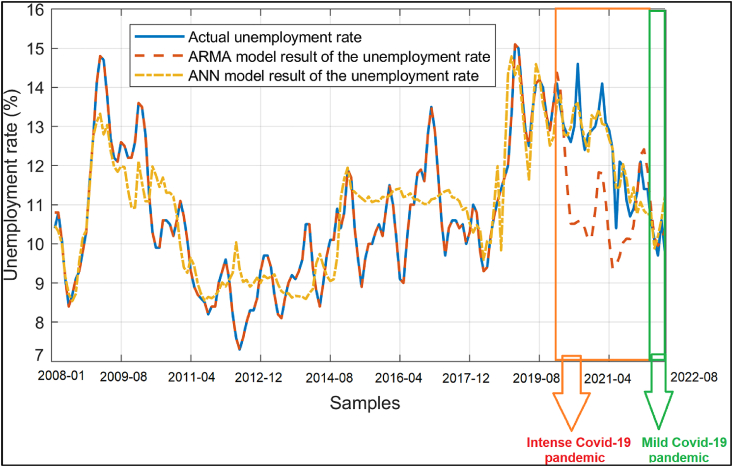
The unemployment rate vs the sample index, actual data (−), result of the ARMA(2, 1) model (**--**) and the result of the ANN model (**--**).

**Fig. 5:**
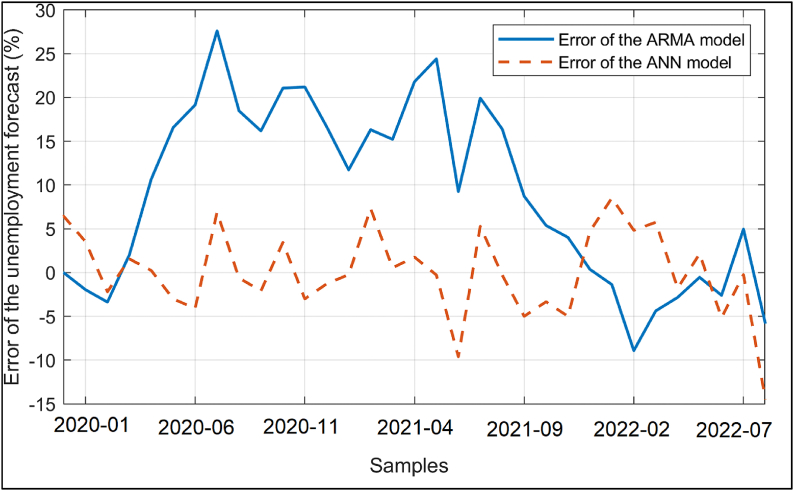
The error percentages of the estimations of the ARMA(2, 1) model (−) and the ANN model (**--**).

The unemployment data and the data obtained from the ARIMA (2,0,1) and ANN models is shown on the same axes in Fig. 4. As it can be seen from Fig. 4, both the model and the feedforward ANN architecture can be used to for the regression of the unemployment rate dependent on the interest rate, exchange rate and oil prices. The input data, output data and the MATLAB function implementing the ANN model with the optimized biases and weights can be accessed from the link given at the end of the paper. The error percentages of the ARMA (2,1) and ANN models are also plotted in Fig. 5. As it can be seen from Fig. 5, the ARMA (2,1) model provides a better model for the unemployment data. For a quantitative comparison of the results of the ARMA (2,1) and ANN models, the mean absolute error (MAE), root mean square error (RMSE), mean absolute percentage error (MAPE) and the coefficient of determination also known as the goodness of fit (R^2^) regarding the ARMA (2,1) and ANN models are also calculated using Eqs. (3)–(3)–(6)(3)–(6) as indicated by Ref. [38] and shown in Table 3. In addition, the last five actual unemployment rate values and the ARMA (2,1) and ANN estimated data are also shown in Table 4 for comparison.

(3) $$MAE=\frac{\sum_{1}^{\dim}\left|O-N\right|}{\dim}$$

(4) $$RMSE=\sqrt{\frac{{\sum_{1}^{\dim}\left(O-N\right)}^{2}}{\dim}}$$

(5) $$MAPE=\frac{100}{\dim}\sum_{1}^{\dim}|\frac{O-N}{N}|$$

(6) $$R^{2}=\frac{\sum_{1}^{\dim}{\left(O-avg\left(O\right)\right)}^{2}-\sum_{1}^{\dim}{\left(O-N\right)}^{2}}{\sum_{1}^{\dim}{\left(O-avg\left(O\right)\right)}^{2}}$$

**Table 3:** Quality metrics of the proposed model results.

Model	MAE	RMSE	MAPE	R^2^
ARMA (2,1)	0.260	0.758	0.020	0.820
ANN	0.718	0.921	0.068	0.735

**Table 4:** Actual unemployment rate and the results of the ARMA (2,1) and the ANN regression models for the last estimation values for 2020–2022 periods.

Date	Data index	Actual Unemployment rate	ARMA (2,1) Estimation	ANN Estimation	Error percentage of the ARMA (2,1)	Error percentage of the ANN model
2020–01	145	14.1	14.37591	13.60176	1.95684%	3.533585%
**2020–02**	**146**	**13.5**	**13.9547**	**13.79631**	**3.368126%**	**2.194907%**
**2020–03**	**147**	**13**	**12.73703**	**12.79706**	**2.022849%**	**1.56104%**
**2020–04**	**148**	**12.8**	**11.43316**	**12.76987**	**10.67841%**	**0.235388%**
**2020–05**	**149**	**12.6**	**10.51231**	**12.97976**	**16.56893%**	**3.013952%**
**2020–06**	**150**	**13**	**10.51136**	**13.53118**	**19.1434%**	**4.086023%**
**2020–07**	**151**	**14.6**	**10.56965**	**13.59409**	**27.60512%**	**6.889789%**
**2020–08**	**152**	**13**	**10.59846**	**13.08056**	**18.47341%**	**0.619672%**
**2020–09**	**153**	**12.4**	**10.39377**	**12.65858**	**16.17929%**	**2.085292%**
**2020–10**	**154**	**12.8**	**10.10325**	**12.35908**	**21.06838%**	**3.444668%**
**2020–11**	**155**	**12.9**	**10.16532**	**13.28796**	**21.19911%**	**3.007459%**
**2020–12**	**156**	**13**	**10.83596**	**13.16615**	**16.64644%**	**1.278042%**
**2021–01**	**157**	**13.4**	**11.82781**	**13.43128**	**11.73277%**	**0.233454%**
**2021–02**	**158**	**14.1**	**11.79949**	**13.06845**	**16.31569%**	**7.315964%**
**2021–03**	**159**	**13.1**	**11.1065**	**13.02909**	**15.21759%**	**0.541306%**
**2021–04**	**160**	**12.9**	**10.08843**	**12.67204**	**21.79514%**	**1.767116%**
**2021–05**	**161**	**12.4**	**9.373185**	**12.43491**	**24.4098%**	**0.281504%**
**2021–06**	**162**	**10.4**	**9.437733**	**11.39912**	**9.252566%**	**9.606888%**
**2021–07**	**163**	**12.1**	**9.689014**	**11.4607**	**19.9255%**	**5.283446%**
**2021–08**	**164**	**12**	**10.03227**	**12.03401**	**16.39774%**	**0.283432%**
**2021–09**	**165**	**11.1**	**10.13183**	**11.65462**	**8.722214%**	**4.996596%**
**2021–10**	**166**	**10.7**	**10.12446**	**11.05655**	**5.378833%**	**3.3322%**
**2021–11**	**167**	**10.9**	**10.46272**	**11.44536**	**4.011761%**	**5.003308%**
**2021–12**	**168**	**11.3**	**11.25849**	**10.76698**	**0.367388%**	**4.716964%**
**2022–01**	**169**	**12.1**	**12.26592**	**11.06926**	**1.371223%**	**8.518553%**
2022–02	170	11.4	12.41631	10.84984	8.914979%	4.825991%
2022–03	171	11.4	11.89902	10.74503	4.37736%	5.745341%
2022–04	172	10.6	10.90041	10.78813	2.834028%	1.774828%
2022–05	173	10.1	10.1541	9.883722	0.535595%	2.141368%
2022–06	174	9.7	9.952305	10.19931	2.601084%	5.147496%
2022–07	175	10.6	10.07336	10.62455	4.968276%	0.231625%
2022–08	176	9.8	10.37176	11.22023	5.834266%	14.49217%

In Eqs. (2)–(2)–(4)(2)–(4), *O* is the original dataset, *N* is the result of the ARMA or ANN forecasts, *dim* is the dimension of the dataset and *avg*() is the arithmetic average function. The MATLAB functions used for the calculation of these parameters are also given as a hyperlink at the end of the paper.

The MAE, RMSE, MAPE and R^2^ values shown in Table 3 indicate that the developed ARMA (2,1) and ANN models have similar performances for the overall period while the ARMA (2,1) model provides slightly better accuracy for the modeling of the unemployment rate dependent on the interest rate, exchange rate and oil prices. In order to further assess the accuracies of the ARMA (2,1) and ANN based forecasts, the Diebold-Mariano tests [39] with and without the Harvey, Leybourne and Newbold (HLN) adjustment [40] are utilized. The mean squared error (MSE) based loss differentials are employed as the standard procedure. The Diebold-Mariano test statistic is computed as 0.471 and 0.312 for the cases with and without the HLN adjustment, respectively. Considering that the Diebold-Mariano test statistic falls inside the range of [−1.96, 1.96] for both the pure and the HLN adjusted cases, it can be concluded that there is no statistically significant difference between the ARMA(2,1) and ANN based separate forecasts for the overall period [41,42] as it is also observed from the R^2^ values given in Table 3. However, it is again worth noting that the ARMA(2,1) model provides slightly better accuracy for the durations outside the Covid-19 pandemic period while the ANN model possesses slightly better precision for the pandemic period as it is observed from Fig. 4 therefore the joint utilization of the ARMA(2,1) and ANN models increases the modeling accuracy for the overall period of 01.2008–08.2022.

Table 4 shows that the unemployment rates and the forecasting error percentages calculated using the ANN and ARMA (2,1) models during and after the Covid-19 pandemic. From Table 4 it is observed that the ANN model outperforms ARMA (2,1) model during the Covid-19 pandemic starting from 2022-02 period.

On the other hand, ARMA (2,1) has a better forecasting accuracy for the post-pandemic period starting from 2022-02. It is worth noting that ARMA (2,1) model already had better predictions before the Covid-19 pandemic as it can be seen from Figs. 4 and 5. Considering these results, the utilization of the ANN models rather than the conventional ARIMA techniques may be recommended in turbulent periods such as the Covid-19 pandemic when the economic uncertainty goes higher.

When the intensity of the Covid-19 pandemic has been lowered after the beginning of the 2022, the forecasting performance of the ARMA (2,1) increases due to the decrease in the economic uncertainty for the 2022:02–2022:08 period. There are several studies in the literature on the forecasting of the unemployment rate using ARIMA modelling techniques. In one of these studies, Katris have shown that there is no globally best method for the forecasting of the unemployment rate and different sampling periods need specific modelling methods [4]. Similarly, Chakraborty et al. have concluded that integrated ARIMA-metaheuristic modelling approaches of the unemployment rate provide better forecasting results when the unemployment data contains linearity and nonlinearity tendencies [5]. Ahmad et al. also studied the utilization of the hybrid ARIMA-metaheuristic modelling methods for the modelling of the unemployment rate of different countries and have concluded that hybrid methods provide better forecasting performance compared to ARIMA-only approaches [6]. Sharma and Soni have also stated that the accuracy of the ARIMA models for the forecasting of the unemployment rate can be improved by integrating the neural network, deep learning and other machine learning approaches [8].

In another study, Katris have demonstrated that the unemployment rates of selected Mediterranean countries can effectively be modelled utilizing hybrid ARIMA and multilayer feed forward neural networks [9]. Comparing the results of our study with the results of these studies, it can be seen that the results of our study is consistent with those of other studies employing hybrid modelling approaches such that the joint modelling approach using linear ARIMA and nonlinear ANN models provides high performance forecasting of the unemployment rate.

According to the results of the study, the unemployment problem in Turkey appears to be chronic and it becomes difficult to predict unemployment with linear models, especially during periods of economic uncertainty. For this reason, the government can make long-term and sustainable labor market regulations for the management of the unemployment. The government should increase its support for on-the-job training, internship and project works, which can adapt to changing conditions in education. Increasing the quality of the workforce and diversifying the flexible trainings suitable for the needs of the enterprises will contribute to the solution of the unemployment problem.

## 4 Conclusions

This study compares the utilization and the performance of the ARIMA and ANN models for modelling the unemployment rate in Turkey for the period of 01.01.2008–31.08.2022. As the first step, the unemployment rate is modelled using a conventional ARIMA model with automatic lag selection leading to ARIMA (2,0,1), that it is an effectively ARMA (2,1) model. In the ARMA model, all of the coefficients obtained for each month of the 12-month period are demonstrated to be statistically significant. This result reveals that it is essential to consider the monthly effects for the modelling of the unemployment rate. Significant monthly effects provide clues in evaluating unemployment as a structural problem. As the next step, the unemployment rate is modelled using an ANN regression model which employs tangent sigmoid functions as the nonlinear activation components. The ANN model is trained using Levenberg-Marquardt method with the random selection of training data to maintain accuracy. The forecasting performances of the ARMA and ANN models are then compared considering the standard evaluation parameters namely RMSE, MAE, MAPE and R^2^. These results are compliant with the estimations given in Refs. [[4], [5], [6],^8^,^9^]. The main result of this study is that the ARMA (2,1) model performs better than the ANN model outside the intense Covid-19 pandemic period while ANN provides a better forecasting performance in this period as shown for the first time in the literature. This result imply that the nonlinear ANN models should be used in conjunction with the conventional autoregressive models for the periods when the economic uncertainty is elevated. In this study, 176 observations which belong to the period of 01.2008–08.2022 are employed for the modeling of the unemployment rate in Turkey. Conventional ARIMA and nonlinear ANN models are used for the forecasting of the unemployment rate using the oil price, interest rate and exchange rate as regressors. The modeling of other key economic parameters such as the energy prices can also be performed employing the joint ARIMA and ANN approach as introduced in this study as the future directions of economic research viewpoint.
