# Supplementary material for: Estimation of the unemployment rate in Turkey: A comparison of the ARIMA and machine learning models including Covid-19 pandemic periods
Source: Heliyon. 2023 Jan 7;9(1):e12796. doi: 10.1016/j.heliyon.2023.e12796 (PMC9860419; doi:10.1016/j.heliyon.2023.e12796)
Supplement: Multimedia component 1 [file mmc1.docx]

**Supplementary Material**

The unemployment rate, oil prices, interest rate and exchange rate data, the developed ANN model and the coefficients of the ARMA (2,1) model can be accessed via the following link: <https://rebrand.ly/2xtfjy6>.
